# Case Report: Clinical characteristics and genetic analysis of two patients with hereditary hemorrhagic telangiectasia

**DOI:** 10.3389/fgene.2022.954796

**Published:** 2022-08-25

**Authors:** Qiu-Ying Wang, Yu-Xuan Feng, Ying-Wei Zhu, Yu-Xia Sun, Jing-Duan Xu, Hui-Min Shi, Yi-Min Mao, Hong-Wei Jiang

**Affiliations:** ^1^ Department of Respiratory Medicine, The First Affiliated Hospital, and College of Clinical Medicine of Henan University of Science and Technology, Luoyang, China; ^2^ Department of Endocrinology, The First Affiliated Hospital, and College of Clinical Medicine of Henan University of Science and Technology, Luoyang, China

**Keywords:** dyspnea, brain abscesses, pulmonary arteriovenous fistula, hereditary hemorrhagic telangiectasia, arteriovenous malformations

## Abstract

**Objective:** To analyze the clinical features and genetic characteristics of two patients with hereditary hemorrhagic telangiectasia (HHT) and to review the relevant literature.

**Methods:** The clinical data of two HHT patients admitted to the author’s hospital between April 2019 and February 2022 were retrospectively analyzed. Meanwhile, the genetic analysis was performed with their consent.

**Results:** The first patient was a 62-year-old woman who had been complaining of shortness of breath and fever for 20 days. Her previous medical history included brain abscess drainage and video-assisted thoracoscopic surgery for a pulmonary hemangioma. A right heart catheterization revealed no pulmonary arterial hypertension, and an abdominal enhanced magnetic resonance imaging revealed multiple arteriovenous malformations in the liver. Her *ACVRL1* heterozygous variants were discovered through whole-exon gene testing. The second case involved a 47-year-old woman who had been experiencing chest tightness for the past 2 years. Several years ago, she underwent brain abscess drainage and embolization of a pulmonary arteriovenous fistula. Ultrasound revealed generalized hepatic vascular dilation, and enhanced computed tomography revealed numerous pulmonary venous fistulas scattered in both lungs as well as multiple arteriovenous malformations in the liver. Her whole-exon gene testing revealed that she, like her son, had heterozygous *ENG* variants.

**Conclusion:** HHT patients may experience infection, bleeding, dyspnea, and other symptoms. Imaging is important in disease diagnosis and management because early detection and treatment can prevent major complications and disability or even death.

## Introduction

Hereditary hemorrhagic telangiectasia (HHT), also called Osler–Weber–Rendu disease, is an autosomal dominant vascular disorder with an estimated prevalence estimates of 1 case per 5,000 people (Faughnan et al., 2020). It is caused most commonly by mutations in *ENG* and *ACVRL1* genes, which affect transforming growth factor beta (TGF-β) signaling (Vorselaars et al., 2017). HHT is characterized by arteriovenous malformations (AVMs), which may present throughout the human body. The disease is diagnosed when three or more of the following Curacao criteria exist: recurrent epistaxis, telangiectasia, visceral lesions, or family history (Faughnan et al., 2011). In China, due to a lack of understanding, HHT is still considered to be a rare disease and is frequently diagnosed late or not at all. In order to review this disease, in this article, we reported clinical characteristics and genetic analyses of two patients diagnosed with HHT in our hospital.

## Cases analysis

### Case 1

**FIGURE 1:**
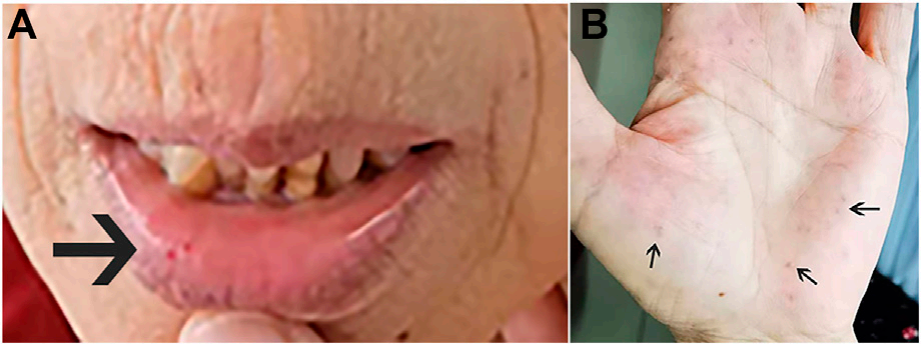
Dilation of the capillary on the oral mucosa **(A)** and the palm **(B)** (black arrow).

**FIGURE 2:**
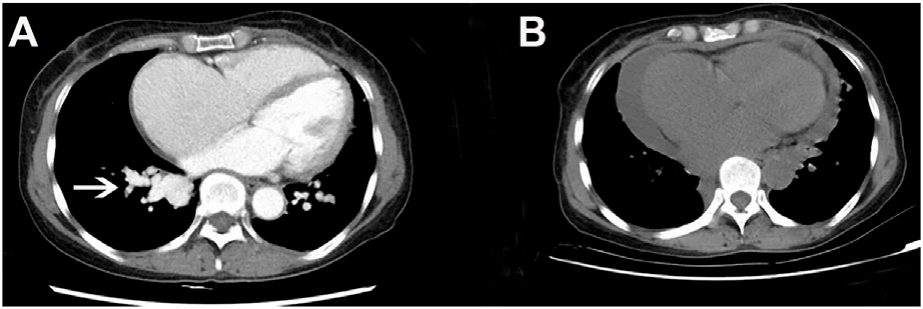
Comparison of chest CT before and after the operation. **(A)** Chest-enhanced CT showed an enhanced vascular mass in the lower part of the right pulmonary artery (white arrow) before operation. **(B)** Plain CT scan of the chest revealed that the former vascular mass had disappeared after the operation.

**FIGURE 3:**
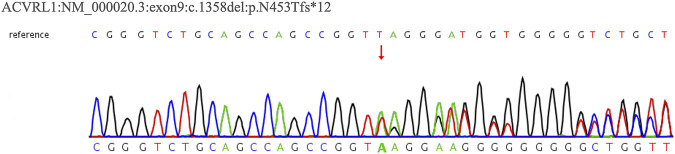
WES result for Case 1’s patient. The sequencing of heterozygous mutation of the *ACVRL1* gene c.1358del.

In February 2022, a 62-year-old woman was admitted to our hospital after complaining of breathlessness and an intermittent fever for more than 20 days. Her previous medical history included brain abscesses and a video-assisted thoracoscopic surgery for pulmonary neoplasm 6 months ago, with a histopathological diagnosis of hemangioma. She remembered having epistaxis episodes and sporadic bleeding 40 years ago, which were mostly triggered or exacerbated by exertion or stress. Physical examination revealed telangiectasias in the oral mucosa and hand (Figure 1), wet rale from the bottom of the left lung, an atrial fibrillation rhythm, and mild edema in both lower limbs. Comprehensive laboratory testing was mostly normal, with the exception of mild anemia, and the assessment of thrombophilia revealed no significant abnormalities except for a higher D-dimer. Transthoracic echocardiography showed an obvious enlargement of the left heart and right atrium, massive tricuspid regurgitation (14.9 cm^2^), and elevated pulmonary artery systolic pressure (42 mmHg), but right heart catheterization indicated no pulmonary arterial hypertension with a mean pulmonary artery pressure of 20 mmHg, a pulmonary capillary wedge pressure of 9 mmHg, and a pulmonary vascular resistance of 2.16 Wood. Chest computed tomography (CT) displayed that compared to the prior CT scan (about 10 months ago, Figure 2), the abnormal dilated vascular mass in the lower lobe of the right lung showed postoperative change. An enhanced magnetic resonance imaging (MRI) of the abdomen revealed multiple hepatic arteriovenous malformations, implying hereditary hemorrhagic telangiectasia. Until that point, we knew that the patient met the clinical criteria of HHT: recurrent, spontaneous epistaxis, mucocutaneous telangiectasias, and a visceral arteriovenous malformation. During this hospitalization, the patient recovered and was discharged on the 20th day after being treated with antibiotics, diuretics, cardiotonic drugs, and other supportive care measures. Meanwhile, we performed the genetic test with her consent. Full exon high-throughput gene sequencing was adopted to detect mutations, and the results were heterozygous variants of the *ACVRL1* gene c.1358del (p.N453T) (Figure 3) in exon9, which has pathogenic significance according to biological analysis. Finally, this patient was genetically diagnosed with HHT2. Before this article was published, the patient was hospitalized again for dyspnea, and during this time, she had a major seizure that lasted about an hour. We performed a cranial MRI but found no clear hemorrhage, and she was cured again without complications through mechanical ventilation and sedation. Regarding her family, one of her sisters and her young daughter both suffered from nasal hemorrhage, but they refused to undergo imaging screening and genetic testing. We advised them to monitor their oral hygiene and fecal status and to seek medical attention as soon as possible.

### Case 2

**FIGURE 4:**
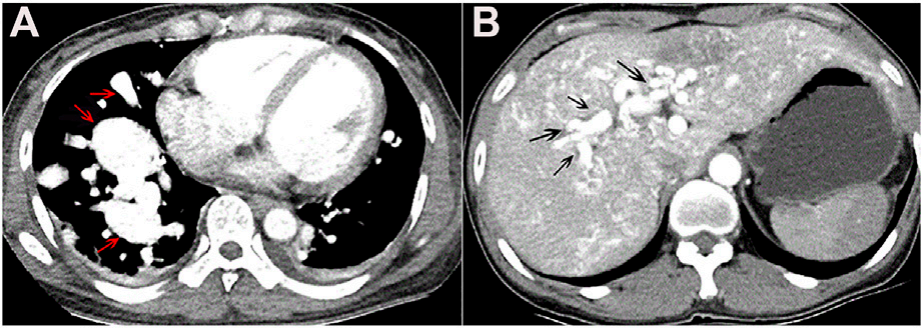
**(A)** Chest-enhanced CT image in the arterial phase showed multiple vascular masses in bilateral lungs, mainly in the lower lobe of the right lung, suggesting pulmonary arteriovenous fistula (red arrow). **(B)** Epigastrium-enhanced CT showed multiple hepatic arteriovenous malformations (black arrow).

**FIGURE 5:**
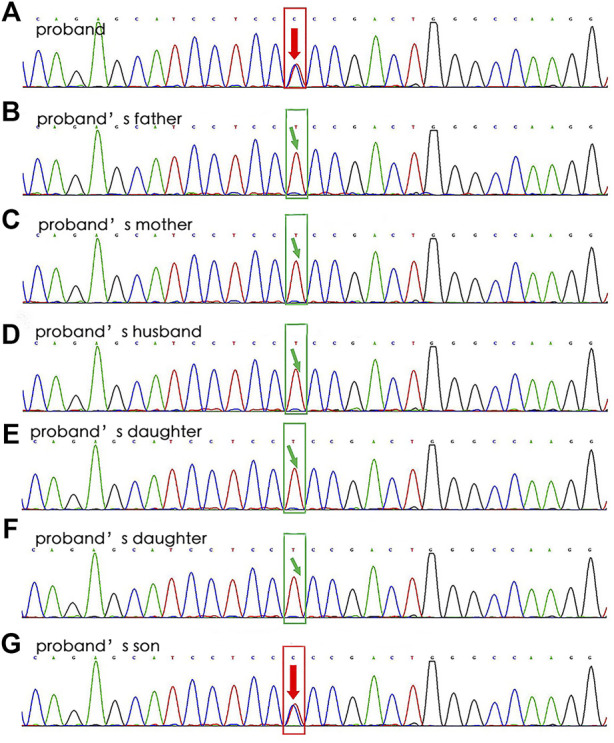
WES results for Case 2’s family. The sequencing of the *ENG* gene c.509T > C (p.Leu170Pro) results in the proband (**A**, heterozygous mutation) and his son (**G**, heterozygous mutation), without in other relatives **(B–F)**.

In April 2019, a 47-year-old female was admitted to our hospital after complaining of chest tightness for 2 years that had worsened with fever for 7 days. She underwent brain abscess drainage at the age of 30 and pulmonary arteriovenous fistula embolization for hemoptysis at the age of 35. As a teenager, she suffered from recurrent epistaxis. A physical examination revealed mild cyanosis of the face and lips, clubbed fingers, and a blowing murmur in the right lower lung. Her blood tests revealed that her arterial blood gas indicated hypoxemia, with PaO_2_ of 58 mmHg on room air and PCT of 12.0 ng/ml, but her other microbiological (including blood, sputum, and urine), biochemical, tumor marker, and antinuclear antibody tests were normal. Abdominal color Doppler ultrasound revealed generalized hepatic vascular dilation. Chest-enhanced CT revealed numerous pulmonary venous fistulas scattered throughout the bilateral lungs (Figure 4A), and abdominal enhanced CT revealed multiple hepatic venous fistulas (Figure 4B). The possibility of HHT is heavily considered in clinical diagnosis when combined with the patient’s medical history. After signing the informed consent form, we performed the genetic test. Full exon high-throughput gene sequencing was adopted to detect mutations by Illumina NovaSep 6,000, and the result showed heterozygous variants of the *ENG* gene c.509T > C (p.Leu170Pro) (Mallet et al., 2015) (Figure 5), which has a pathogenic significance according to biological analysis. Concurrently, the same heterozygous variant was found in her son. (Figure 5). Finally, this patient was genetically diagnosed with HHT1. During the hospitalization, the patient felt alleviated after receiving antibiotics, an oxygen supplement, and other supportive care measures and was discharged on the 15th day. Unfortunately, due to financial constraints, she declined the interventional embolization of pulmonary arteriovenous fistula. Prior to that, she was lost to follow-up 3 years later. We tried our best to visit her home and see her husband and her son before this article was published unfortunately, but the patient died of massive hemoptysis 2 years before. Moreover, because her son had mild epistaxis twice, we advised him to pay attention to dental hygiene and antibiotic prophylaxis before dental care as well as to have blood tests and imaging screening on time.

### Genetic analysis

Whole-exome sequencing (WES) revealed that Case 1’s patient had a heterozygous variant of the *ACVRL1* gene: NM_000020.3:exon9:c.1358del (p.N453T) (Figure 7). According to the ACMG guideline, the variant was judged to be likely pathogenic (PVS1_strong + PM2), and it was not included in the ClinVar database. Regrettably, we cannot get a further proving of the source without the consent of her family members.

WES results for a patient of Case 2’s family revealed that she had a heterozygous variant of the *ENG* gene: NM_000118.3: c. T509C (p. L170P) ^4^ (Figure 8). A heterozygous mutation was detected in the exonic region of the *ENG* gene c.509T > C, resulting in an amino acid change: p. L170P. The mutation site was reported as a pathogenic variant in Human Gene Mutation Database, PMID:2,5312062, but was not included in the ClinVar database. By genetic analysis of her family members, the patient and her son were consistent with the same phenotype of the disease, and she was most likely to have a *de novo* mutation because neither of her parents has it.

## Discussion

HHT is an autosomal dominant vascular disorder, with an estimated prevalence of 1 case per 5,000–10,000 individuals in North America (Faughnan et al., 2020; Ferry et al., 2020). However, there is no exact incidence rate in China because the disease is still considered rare and most studies are case reports (Lin et al., 2001; Zhang et al., 2004; Lu et al., 2020; Xu et al., 2021). Because of the variety of clinical manifestations, HHT is frequently misdiagnosed, and most patients may have a long medical history before being correctly diagnosed (Lu et al., 2011; Kritharis et al., 2018). According to a recent study, about 26.4 ± 17.0 years was delayed from the onset of HHT-related clinical signs or symptoms to the correct diagnosis of HHT in China (Li et al., 2018). Patients may seek medical advice from the E.N.T. department for epistasis, the neurosurgery department for brain abscess, the hematology department for anemia, the respiratory department for hypoxia, and so on. Like this report, both patients had epistaxis, a brain abscess, and a pulmonary vascular malformation after being diagnosed with HHT.

HHT is a clinical diagnosis based on the following Curaçao criteria (Faughnan et al., 2011; Faughnan et al., 2020): spontaneous and recurrent epistaxis, telangiectasias, visceral arteriovenous malformations, or a first degree relative conformed HHT. Patients who met three to four criteria were definitely diagnosed with HHT, those who met two criteria were probably diagnosed with HHT, and those who met 0–1 criteria were unlikely to have HHT. Epistaxis is considered to be the most common clinical characteristic, having been reported in up to 95% of HHT patients (Kritharis et al., 2018; Li et al., 2018; Ferry et al., 2020). However, large arteriovenous malformations (AVMs) can occur in the lungs, brain, and liver, resulting in a stroke, brain abscess, and seizure, respectively (Kritharis et al., 2018). Pulmonary AVMs, frequently undiagnosed and asymptomatic, are observed in 15–60% of patients (Lacombe et al., 2013; Kritharis et al., 2018; Li et al., 2018). Modern screening techniques detect hepatic AVMs in more than 70% of patients, but only 8% of them develop symptomatic liver disease. Cerebral AVMs are less frequent (10–23% of HHT patients), but their consequences can be fatal (Kritharis et al., 2018; Lu et al., 2020).

HHT is an autosomal dominant genetic disease caused by monoallelic mutations in the genes *ENG* (HHT1), *ACVRL1* (HHT2), and *SMAD4* (HHT3) and other uncommon genes such as *BMP9/GDF2, RASA-1*, and *EPHB4*. More than 80% of HHT patients have identifiable genetic mutations, and over 90% of mutations are observed in either the *ENG* (61%) or *ACVRL1* (37%) gene of the genetically screened patients (Kritharis et al., 2018). Interestingly, the *ACVRL1* gene variant is over twice more prevalent than that in *ENG* in Chinese patients with HHT (Zhao et al., 2019). HHT1 is caused by mutations in the ENG gene on chromosome 9, which encodes the protein endoglin, whereas HHT2 is caused by mutations in the *ACVRL1* gene on chromosome 12, which encodes the protein ALK-1, and HHT3 is caused by mutations in the *MADH4* gene on chromosome 5, which encodes the SMAD4 protein (Vorselaars et al., 2017; Kühnel et al., 2018). All the identified genes are involved in the TGF-β/BMP signaling pathway, which can regulate cell growth, apoptosis, and vascular remodeling and maintenance (Shovlin, 2010). It was reported that certain mutated genes in HHT may be linked to specific clinical manifestations. *ENG* mutations (HHT1) are more likely to cause pulmonary and brain AVMs; 5–30% of patients with pulmonary AVMs may be asymptomatic or present with hemoptysis and dyspnea. Noteworthily, migraines are not uncommon in patients with pulmonary AVMs. Infections of the central nervous system, such as brain abscesses, may occur in 1% or more of HHT patients, ranging in severity from mild to life-threatening. It has been proposed that bacterial seeding or septic emboli may travel from pulmonary AVMs to the brain (Dong et al., 2001; Kritharis et al., 2018). According to studies, *ACVRL1* (HHT2) mutation is correlated with an increased risk of liver AVMs, spinal AVMs, epistaxis, and pulmonary hypertension (Vorselaars et al., 2017; Latif et al., 2021). The majority of patients with liver AVMs are asymptomatic, but shunting blood through these AVMs can cause high-output heart failure, liver failure, or portal hypertension. Pulmonary hypertension (PH) is increasingly thought to be a potentially fatal complication of HHT because it is associated with a poor prognosis and can lead to progressive right heart failure and death (Galiè et al., 2015). Shunting of blood from the hepatic arteries or portal veins to the hepatic veins caused a hyperdynamic state in patients with hepatic AVMs, with the cardiac output (CO) increasing up to threefold (Garcia-Tsao et al., 2000; Chen et al., 2020). Abston et al. (Abston et al., 2021) reported that pulmonary vasodilator therapy may be an available treatment to improve hemodynamics in HHT with PAH patients.

Because there have been few randomized trials, no standard medical therapies have been recommended for HHT. Options for treatment are patient-specific and are best categorized according to local versus systemic measures. The treatment criterion is composed of supportive care, lesion-specific therapy, and systemic therapy. Treatment involving otolaryngology, interventional radiology, or neurosurgery was considered lesion-specific therapy (Kritharis et al., 2018). The size, location, and symptoms of an AVM may influence therapeutic measures such as pulmonary AVM embolization, surgical intervention for a CNS AVM, and/or continued surveillance. In the vast majority of HHT patients, percutaneous embolization of pulmonary AVMs may be a safe, effective, and long-term treatment option (Lacombe et al., 2013; Hetts et al., 2021; Kolarich et al., 2021). Interventional radiologists, on the other hand, consider angiographic treatment for hepatic AVMs to be a high-risk procedure, whereas liver transplantation can reduce mortality, particularly in patients with high output cardiac failure (Iyer et al., 2019; Hetts et al., 2021). Bevacizumab, a recombinant humanized monoclonal antibody that blocks angiogenesis by inhibiting the vascular endothelial growth factor (VEGF), appears promising in HHT treatment as an intravenous method of reducing the frequency and severity of epistaxis and improving quality of life (Riss et al., 2015). However, data on intranasal bevacizumab were contradictory as studies on intravenous bevacizumab use were limited to case reports or retrospective series (Iyer et al., 2018). Pazopanib is an orally administered tyrosine kinase inhibitor that can block VEGF receptors and thus act as an anti-angiogenic treatment for HHT. There was an improvement in anemia and/or epistaxis in HHT patients, but no serious adverse events occurred (Faughnan et al., 2019). Thalidomide was used as an anti-VEGF therapy drug to treat HHT patients who had gastrointestinal bleeding or epistaxis (Bauditz et al., 2004; Lebrin et al., 2010; Li et al., 2018). Other immunomodulatory drugs such as tacrolimus have been used to treat patients with HHT and pulmonary arterial hypertension (Sommer et al., 2019).

## Conclusion

HHT is a rare autosomal dominant vascular disorder caused by mutations in the *ENG* and *ALK1* genes. Imaging may be useful in the diagnosis and management of this disease, particularly in the case of arteriovenous malformations in the brain, lungs, or liver. With early diagnosis, followed by adequate screening and treatment, the major complications of this disorder can often be avoided and disability or even death can be prevented.

## Data Availability

The data sets for this article are not publicly available due to concerns regarding participant/patient anonymity. Requests to access the data sets should be directed to the corresponding author.
